# Understanding the association between psychomotor processing speed and white matter hyperintensity: A comprehensive multi‐modality MR imaging study

**DOI:** 10.1002/hbm.24826

**Published:** 2019-11-01

**Authors:** Shuyue Wang, Yeerfan Jiaerken, Xinfeng Yu, Zhujing Shen, Xiao Luo, Hui Hong, Jianzhong Sun, Xiaojun Xu, Ruiting Zhang, Ying Zhou, Min Lou, Peiyu Huang, Minming Zhang

**Affiliations:** ^1^ Department of Radiology The Second Affiliated Hospital of Zhejiang University School of Medicine Hangzhou China; ^2^ Department of Neurology The Second Affiliated Hospital of Zhejiang University School of Medicine Hangzhou China

**Keywords:** cortex, diffusion tensor imaging, functional magnetic resonance imaging, processing speed, tractography, white matter hyperintensity

## Abstract

Cognitive processing speed is crucial for human cognition and declines with aging. White matter hyperintensity (WMH), a common sign of WM vascular damage in the elderly, is closely related to slower psychomotor processing speed. In this study, we investigated the association between WMH and psychomotor speed changes through a comprehensive assessment of brain structural and functional features. Multi‐modal MRIs were acquired from 60 elderly adults. Psychomotor processing speeds were assessed using the Trail Making Test Part A (TMT‐A). Linear regression analyses were performed to assess the associations between TMT‐A and brain features, including WMH volumes in five cerebral regions, diffusivity parameters in the major WM tracts, regional gray matter volume, and brain activities across the whole brain. Hierarchical regression analysis was used to demonstrate the contribution of each index to slower psychomotor processing speed. Linear regression analysis demonstrated that WMH volume in the occipital lobe and fractional anisotropy of the forceps major, an occipital association tract, were associated with TMT‐A. Besides, resting‐state brain activities in the visual cortex connected to the forceps major were associated with TMT‐A. Hierarchical regression showed fractional anisotropy of the forceps major and regional brain activities were significant predictors of TMT‐A. The occurrence of WMH, combined with the disruption of passing‐through fiber integrity and altered functional activities in areas connected by this fiber, are associated with a decline of psychomotor processing speed. While the causal relationship of this WMH‐Tract‐Function‐Behavior link requires further investigation, this study enhances our understanding of these complex mechanisms.

**Abbreviations**
AF: arcuate fasciculus
ATR: anterior thalamic radiations
Cab: cingulum—angular bundle
Ccg: cingulum—cingulate gyrus bundle
CST: corticospinal tract
DTI: diffusion tensor imaging
FA: fractional anisotropy
fALFF: fractional amplitude of low‐frequency fluctuations
FDR: false discovery rate
FMajor: corpus callosum—forceps major
FMin: corpus callosum—forceps minor
GM: gray matter
ILF: inferior longitudinal fasciculus
MMSE: mini‐mental state examination
MNI: Montreal Neurological Institute
MoCA: Montreal Cognitive Assessment
rs‐fMRI: resting‐state functional magnetic resonance imaging
SLFp: superior longitudinal fasciculus—parietal branch
SLFt: superior longitudinal fasciculus—temporal branch
TMT‐A: Trail Making Test Part A
TRACULA: TRActs constrained by UnderLying Anatomy
UNC: uncinate fasciculus
VBM: voxel‐based morphormetry
WM: white matter
WMH: white matter hyperintensity
WMHV: white matter hyperintensity volume

## 1 INTRODUCTION

Psychomotor processing speed describes the amount of time taken to process a set of cognitive operations (Miyake et al., 2000). It is crucial for human brain cognition, and it declines with age (Burgmans et al., 2011; Lu et al., 2011; Morgan, 2007; Voineskos et al., 2012). Slower psychomotor processing speeds can significantly impair daily activities (Pantoni, 2010). Several studies have revealed that decline in processing speed is associated with white matter hyperintensity (WMH), a common symptom in the elderly population (Atwi et al., 2018; Wright et al., 2008). The WMH appears hyper‐intense patches in the white matter on T2 and T2FLAIR images (Fazekas et al., 1993; Wardlaw et al., 2013), and is vascular originated (Wardlaw, Smith, & Dichgans, 2019). While WMH may slow down the processing speed, its intermediate mechanisms have not been fully defined.

The association between WMH and processing speed decline are well studied and the WMH distribution pattern is important. Extended distributions of WMH around the anterior and posterior periventricular horn region, as opposed to deep white matter areas, are associated with executive dysfunction (Lampe et al., 2019; Smith et al., 2011; Sudo et al., 2012; Sun et al., 2017). This phenomenon can be explained by WMH in different locations that are associated with white matter damage on different fibers. Further studies found that FA changes in the anterior thalamus radiata, corpus callosum, and cingulum are related to impaired executive functions in patients with cerebral small vessel disease (Bender, Völkle, & Raz, 2016; Ghanavati et al., 2018; Johnson et al., 2017; Papma et al., 2014; Tuladhar et al., 2015). Based on these findings, it has been proposed that slower processing speeds caused by WMH are a result of “disconnection,” arguing that disrupted fiber integrity slows information flow among different brain regions, leading to brain activity alterations. Indeed, brain functional changes contribute to processing speed decline (Fjell et al., 2017; Jacobs et al., 2012; Langen et al., 2018; Madden et al., 2017; O'Sullivan et al., 2001; Seiler et al., 2018; Shenkin et al., 2005; Tuladhar et al., 2016). Whether this is caused by fiber disruption or gray matter damage remains undefined.

Previous studies implicated a possible association between WMH, white matter tract, and brain function and behavior, suggesting that WMH contributes to brain cognition decline by damaging passing‐through fibers and altering brain activities in regions connected by the fibers. Indeed, severe fiber demyelination is observed in WMH areas (Muñoz Maniega et al., 2019). As the majority of brain function relies on efficient information transmission through brain fibers, its microstructural integrity directly affects information processing in the connected cortex (O'Sullivan et al., 2001), and may also cause retrograde neuron degeneration (Duering et al., 2015; Tuladhar et al., 2015). While this WMH‐Tract‐Function‐Behavior link aligns with our neuroscience knowledge, further supporting evidence is required. Previous studies explored this link by analyzing the relationship between processing speed and one or two brain features (Dhamoon et al., 2018; Hirsiger et al., 2017; Lampe et al., 2017; Marquine et al., 2010; Seiler et al., 2018; Smith et al., 2011; Tuladhar, Reid, et al., 2015; Tullberg et al., 2004), and the results were inconsistent (Madden et al., 2017; Seiler et al., 2018; Shi et al., 2017). Alterations that are specifically related to spatial relationships of brain structural and functional changes have not been unified.

This study had two primary goals. The first was to fully demonstrate whether WMH, fiber integrity, and gray matter alterations are associated with psychomotor processing speed changes. To achieve this goal, we included the analyses of macrostructure (distribution patterns of WMH and regional gray matter volume), microstructure (fiber integrity), and brain resting‐state activities. We used the Trail Making Test part A (TMT‐A) assess psychomotor processing speed. Due to the involvement of the speed of processing, attention, visual scanning and search, number recognition, numeric sequencing, TMT‐A was believed to assess the cognitive domains including processing speed mental flexibility and visual‐motor skills and has been widely used to evaluate psychomotor processing speed in healthy subjects and patients (ter Telgte et al., 2018). Our previous study has also demonstrated that TMT‐A completion time was related to WMH severity (Luo et al., 2017). Our second goal was to validate the WMH‐Tract‐Function‐Behavior link, by observing whether an overlapping pattern among results derived from different modalities exists. As previous studies have shown inconsistent results, we did not select priori regions or tracts, allowing us to capture the spatial patterns of focal pathology that could be more general and universal.

## 2 SUBJECTS AND METHODS

### 2.1 Subjects

The study was approved by the Medical Ethics Committee of the Second Affiliated Hospital, Zhejiang University School of Medicine. The written informed consents were obtained from all subjects.

We retrospectively reviewed patients admitted to the Department of Neurology who received brain MRI and were diagnosed with cerebral small vessel disease between December 2015 and December 2017. Those patients had no specific symptoms but were found to have WMH lesions, which indicate mild alterations in brain vascular systems. Inclusion criteria were as follows: (a) visible WMHs on T2 FLAIR; (b) Fazekas score of WMHs >2; (c) age > 40; (d) normal vision and hearing. Exclusion criteria were as follows: (a) WM lesions of non‐vascular origin (immunological‐demyelinating, metabolic, toxic, infectious, etc.); (b) severe head motion during MRI scanning; (c) history of stroke, multiple sclerosis, Alzheimer's disease, Parkinson's disease, and head trauma.

**Figure 1:**
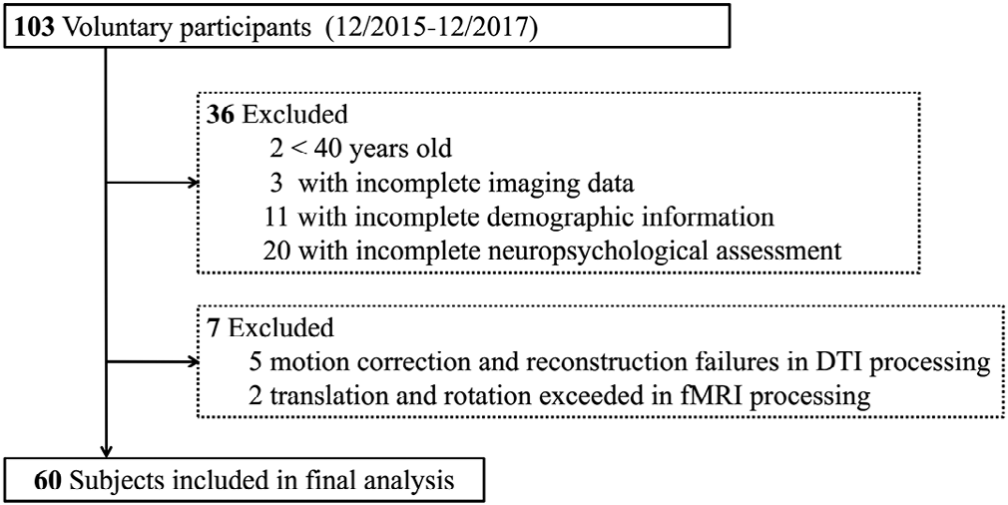
Subject selection flowchart. Patients with WMHs were reviewed between December 2015 and December 2017 from the Department of Neurology. A total of 60 subjects were included in after hierarchical exclusion. WMHs, white matter hyperintensities

Sixty subjects were included after hierarchical exclusion. Exclusion details are shown in Figure 1. Demographic information and vascular risk factors including age, gender, diabetes, hypertension, hyperlipidemia, heart disease, smoking, and drinking histories were recorded in Table 1.

**Table 1:** Clinical and demographic data of participants

	*N* = 60
Age, years, mean (*SD*)	64.8 (9.6)
Males (%)	30 (50)
Diabetes (%)	9 (15.0)
Hypertension (%)	37 (61.7)
Hyperlipidemia (%)	2 (3.3)
History of heart disease (%)	2 (3.3)
History of smoking (%)	16 (26.7)
History of drinking (%)	15 (25.0)
MMSE (*SD*, range)	26.9 (2.6, 17–30)
MoCA (*SD*, range)	23.5 (3.4, 14–30)
TMT‐A completion time (*SD*, s)	82.22 (34.90)
WMHV (*SD*, cm^3^)	21.5 (16.4)
Frontal WMHV (*SD*, cm^3^)	7.98 (7.82)
Temporal WMHV (*SD*, cm^3^)	1.89 (2.01)
Parietal WMHV (*SD*, cm^3^)	7.60 (6.43)
Occipital WMHV (*SD*, cm^3^)	1.12 (1.16)
Subcortical WMHV (*SD*, cm^3^)	4.20 (2.61)
ICV (*SD*, cm^3^)	1,423.1 (141.8)

*Note*: Data are presented as mean (*SD*, range) or number (percentage).

Abbreviations: ICV, intracranial volume (cm^3^); MMSE, mini‐mental state examination; MoCA, Montreal Cognitive Assessment; TMT‐A, Trail Making Test Part A (second); WMHV, white matter hyperintensity volume (cm^3^).

### 2.2 Neuropsychological assessment

The neuropsychological condition of each subject was assessed by the mini‐mental state examination (MMSE), Montreal Cognitive Assessment (MoCA). Psychomotor processing speeds were evaluated with TMT‐A (Bowie & Harvey, 2006). In part A, all the participants used a pencil to connect a series of 25 encircled numbers in numerical order under the standard administration instructions. The completion time is evaluated by scoring the time in seconds, and the maximum completion time for TMT‐A was limited to 180 s.

### 2.3 MRI data acquisition

All subjects experienced multi‐model MRI on a 3.0 T MR (MR750, GE Healthcare) scanner using an 8‐channel brain phased array coil. Scanning modalities included high‐resolution 3D sagittal T1‐weighted imaging (3DT1), T2 FLAIR imaging, diffusion tensor imaging (DTI) and resting‐state functional magnetic resonance imaging (rs‐fMRI). 3DT1 was acquired using spoiled gradient echo sequences with TR/TE = 7.3/3.0 ms, TI = 450 ms, flip angle = 8°, slice thickness = 1 mm, matrix = 250 × 250, field of view (FOV) = 250 mm × 250 mm. The sequence parameters of T2 FLAIR were: TR/TE = 8,400/152 ms, TI = 2,100 ms, flip angle = 90°, slice thickness = 4 mm without slice gap, matrix size = 256 × 256, FOV = 240 mm × 240 mm. DTI was performed with a single shot, diffusion‐weighted spin echo echo‐planar imaging sequence. Maximum *b*‐values were 1,000 s/mm^2^ in 30 noncollinear directions; 5 volumes were acquired without diffusion weighting (*b*‐value = 0 s/mm^2^). Other parameters of DTI were as follows: TR/TE = 8,000/80.8 ms, flip angle = 90°, slice thickness = 2 mm without slice gap, matrix size = 128 × 128, FOV = 256 × 256. Rs‐fMRI was acquired using a gradient recalled echo/echo planar imaging sequence and comprised a time series of 180 volumes with the following parameters: TR/TE = 2,000/30 ms; flip angle = 77°; FOV = 240 mm × 240 mm; matrix size = 64 × 64; slice thickness = 4 mm; slice gap = 1 mm; slices = 38.

### 2.4 Image processing and analysis

#### 2.4.1 WMH lesion distribution analysis

Based on our previous studies, WMH volumes in different regions, as opposed to whole brain WMH volumes, were specifically related to psychomotor speed reduction. We initially assessed WMH volumes in five cerebral ROIs. The detailed procedure of WMH segmentation has been described in our previous studies (Jiaerken et al., 2019; Luo et al., 2017). In general, 3DT1 image and T2 FLAIR images of each subject were used to automatically segment WMH lesions using the Lesion Segmentation Toolbox in SPM12 (Schmidt et al., 2012). All voxels in the 3DT1 images were labeled as gray matter, white matter, or cerebrospinal fluid. The hyper‐intense regions of each tissue class were extracted based on the T2 FLAIR images. Experiencing an iterative algorithm, the lesion expanded based on voxel‐wise weighted assumption and the anatomical WM location. The segmented results were visually inspected and manually corrected in ITK‐SNAP software (http://www.itksnap.org).

We co‐registered the standard atlas (UNC adult brain atlas template, created by University of North Carolina at Chapel Hill, http://www.nitrc.org) to the 3DT1 images of each subject. The UNC lobar parcellation mask had five different ROIs, encoding the frontal, occipital, temporal, parietal lobe, and subcortical region. The 3DT1 image was co‐registered to T2 FLAIR and the deformation field was applied to the registration of individual cerebral masks to T2 FLAIR images. These cerebral masks were used to extract the WMH volume in five cerebral ROIs by combing WMH lesion maps.

#### 2.4.2 VBM analysis

VBM was used to assess the relationship between regional gray matter volume and processing speed, and was implemented by the Statistical Parametric Mapping toolbox (SPM12, http://www.fil.ion.ucl.ac.uk/spm). Processing steps included: (a) Segment T1 weighted images into gray matter, white matter, and cerebrospinal fluid (CSF) using the unified segmentation module. (b) Study‐specific group templates using Diffeomorphic Anatomical Registration using Exponentiated Lie algebra (DARTEL) (Ashburner, 2007), through which we could achieve accurate inter‐subject registration. (c) Normalization of each participant's brain into the MNI space, using the group brain template as the intermediate image. Normalized images were modulated to ensure that the relative gray matter volumes were preserved following spatial normalization. (d) Modulated GM images were smoothed with a 6 mm FWHM Gaussian kernel filter. Two specialized radiologists visually checked the output results in addition to the correction of segmentation errors based on corresponding WMH lesion masks.

#### 2.4.3 Fiber tract analysis

To explore the relationship between white matter integrity and psychomotor speed, we used TRACULA (TRActs Constrained by UnderLying Anatomy) (Yendiki, Reuter, Wilkens, Rosas, & Fischl, 2016), a probabilistic automatic tracking method based on Freesurfer (version 6.0, http://surfer.nmr.mgh.harvard.edu/fswiki) and FSL (https://fsl.fmrib.ox.ac.uk/fsldownloads), to perform fiber reconstruction. TRACULA utilizes the underlying anatomic pathways from the fiber tracts atlas of the training datasets and is capable of reconstructing 18 major white matter tracts.

The workflow and technical details are described in prior publication (Lee et al., 2015). In general, (a) Structural segmentation: Cortical parcellation and subcortical segmentation of raw T1‐weighted data were implemented in FreeSurfer. The structure segmentation results of each subject, covering the locations and orientations of tracts, were required as anatomical references for tract tracking; (b) Pre‐processing of DTIs: A standard pre‐processing method for DTI, including eddy currents and motion correction, was performed by registering the diffusion‐weighting to the *b* = 0 images. Tensor fitting was used for extraction of tensor‐based measures (i.e., FA, MD); (c) Reconstruction: the FSL's bedpostX algorithm which is based on a “ball‐and‐stick” diffusion model was used to calculate the model parameters of each voxel (Behrens et al., 2003; Behrens, Berg, Jbabdi, Rushworth, & Woolrich, 2007). To ensure the pre‐processing quality for diffusion MRIs, the head motion index computed from the affine registration was evaluated in the TRACULA report (Yendiki, Koldewyn, Kakunoori, Kanwisher, & Fischl, 2014). Two subjects were excluded due to abrupt translation and rotation. By combining the anatomic structure segmentation of each subject's T1 imaging, the probabilistic distribution of the pathways was reconstructed.

**Figure 2:**
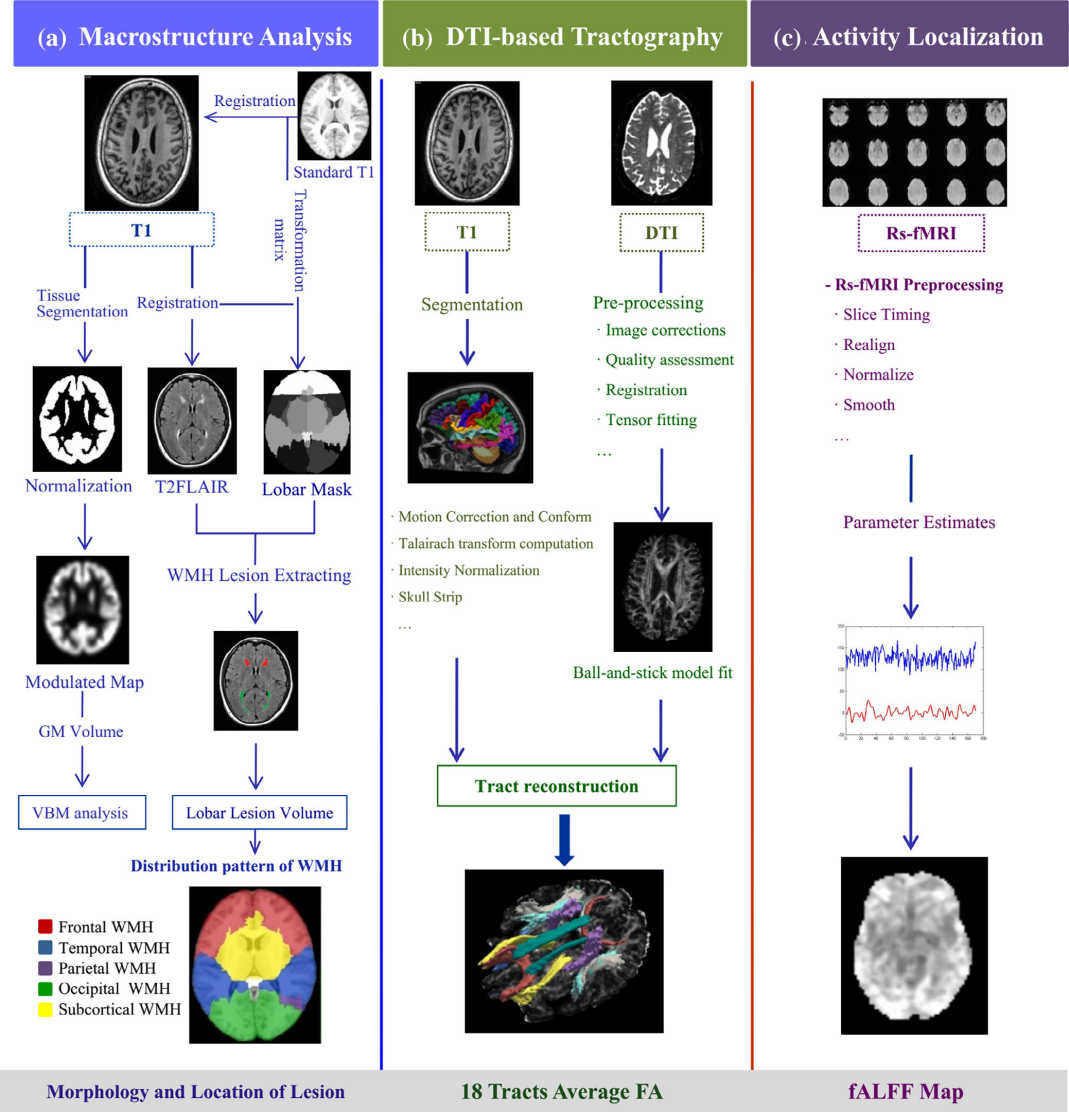
Workflow of multi‐modality imaging pre‐processing. (a) Macrostructure analysis. 3DT1 and T2 FLAIR images were used for the analysis of WMH lesions. 3DT1 images were normalized to the modulated maps following tissue segmentation. We co‐registered the standard atlas to the 3DT1 image of each subject. The 3DT1 image was co‐registered to T2 FLAIR, and the deformation field was obtained for the registration of individual cerebral masks to T2 FLAIR images. The final cerebral masks in the subject's T2 FLAIR space were used to extract the WMH volume in five cerebral ROIs through combing with the WMH lesion map; (b) DTI‐based tractography. The cortical parcellation and subcortical segmentation of raw 3DT1 data and pre‐processing of DTIs were implemented. The probabilistic distribution of the pathways was reconstructed by combining the tracts atlas and labeled structure segmentation data from each subject in the native diffusion space; (c) Activity localization. Rs‐fMRI was used to obtain the fALFF values, and the coefficient map of each subject was normalized to z‐scores across the whole brain after pre‐processing. DTI, diffusion tensor imaging; fALFF, fractional amplitude of low‐frequency fluctuations; GM, gray matter; Rs‐fMRI, resting‐state functional magnetic resonance imaging; WMH, white matter hyperintensity

Finally, 18 WM pathways were reconstructed from each subject (Figure 2), including the corticospinal tract (CST), uncinate fasciculus (UNC), inferior longitudinal fasciculus (ILF), anterior thalamic radiations (ATR), cingulum—cingulate gyrus bundle (Ccg), cingulum—angular bundle (Cab), superior longitudinal fasciculus—parietal branch (SLFp), superior longitudinal fasciculus—temporal branch (SLFt), corpus callosum—forceps major (FMajor), and corpus callosum—forceps minor (FMin). Except for the corpus callosum, all other pathways were reconstructed for the left (L) and right (R) hemisphere. The average FA of each tract was obtained.

Each subject's reconstructed tracts underwent visual inspection, and aberrant or truncated fiber tracts were excluded. Five subjects were excluded due to motion artifacts and reconstruction failures, leaving 62 cases for subsequent analyses.

#### 2.4.4 Resting‐state functional MRI

The processing of raw rs‐fMRIs was performed using the Statistical Parametric Mapping toolbox (SPM12, http://www.fil.ion.ucl.ac.uk/spm) and the Data Processing Assistant for Resting‐State fMRI toolbox (DPARSF, http://www.rfmri.org). The initial 10 time points of each rs‐fMRI scan were removed to eliminate the effects of signal non‐equilibrium and unsteady states. The remaining rs‐fMRI data underwent slice timing to correct acquisition delays. Then, the functional volumes for each subject were motion‐corrected using rigid‐body transformation to align all BOLD images to the first volume (Yan, Wang, Zuo, & Zang, 2016). Data were discarded if the translation exceeded 2 mm or if the rotation exceeded 2^°^. Two subjects were excluded under these criteria. The corrected rs‐fMRI images were co‐registered to the standard Montreal Neurological Institute (MNI) space via T1‐weighted images from each subject, resampled to the 3‐mm isotropic voxels and spatially smoothed using 6 × 6 × 6 mm^3^ Gaussian kernels. Nuisance covariate regression was used to regress out the head motion using the 24 motion parameters from the imaging realignment estimation results (Friston, Williams, Howard, Frackowiak, & Turner, 1996; Satterthwaite et al., 2013; Yan et al., 2013). To regress out physiological noises, T1‐weighted image was segmented into WM and CSF masks (Ashburner & Friston, 2005). Then the CompCor method was applied to extract the first five principal components from a combined WM/CSF mask which were regressed out in the step of nuisance covariate regression (Behzadi, Restom, Liau, & Liu, 2007).

The pre‐processed functional data were used for fALFF calculations at each intracranial voxel. The Fractional amplitude of low‐frequency fluctuation (fALFF) representing the ratio of the sum of amplitudes of low‐frequency (0.01–0.08 Hz) to the sum of Fourier amplitudes across the entire frequency range (Zou et al., 2008), was calculated using the pre‐processed functional data. Specifically, the pre‐processed time series for each voxel was first transformed to the frequency domain using a Fast Fourier Transform (FFT), and the power spectrum was obtained. The square root of the power spectrum was computed at each frequency of the power spectrum, and the averaged square root was obtained across the low‐frequency (0.01–0.08 Hz) at each voxel. Then, fALFF value was calculated as the ratio of the low‐frequency power spectrum to the power spectrum of the entire frequency range. Finally, the generated fALFF coefficient map of each subject was normalized to z‐scores across the whole brain, which was used to the subsequent analyses.

### 2.5 Statistical analysis

All numerical data were analyzed using SPSS version 23.0 (SPSS, Inc., Chicago, IL). To examine the relationship between WM lesions and psychomotor processing speed changes, linear regression analyses were performed between the TMT‐A completion time and WM imaging features, including WMH volumes in five cerebral ROIs and the average FA values from 18 reconstructed tracts, controlling for age and gender.

TMT‐A completion times were set as the dependent variable. Variables that reached a *p* value of <.05 were selected as inputs for further hierarchical multiple regression models to estimate significant predictors of psychomotor processing speed.

To investigate the association of psychomotor processing speed with regional gray matter volume and brain activity, voxel‐wise linear regression analyses were performed between the TMT‐A completion time and brain maps (modulated gray matter map and fALFF maps) in each voxel, controlling for age and gender. Significant result was corrected by false discovery rate (FDR) method, controlling for age and gender (*p* < .05). After voxel based comparisons across whole brain, the mean fALFF values were extracted from significant clusters for further analysis.

Based on these findings, significant variables of structural‐functional indices were entered into hierarchical multiple regression analysis in a forward fashion, so that variables were only entered if they statistically improved the model. TMT‐A completion times were set as the dependent variable. Age was entered into the initial block. Occipital WMH volumes, FA in tracts (FA in FMajor, right Cab, and right UNC) and regional fALFF values were added into further blocks in a stepwise forward fashion. Adjusted *R* ^2^ (explanation of variance), incremental explanation of variance (Δ), standardized beta values (*βj*) and the *p* values of the change in variance between the models were calculated.

## 3 RESULTS

### 3.1 Demographic and clinical data

Sixty subjects were included in the final analysis. Detailed demographic and clinical data are presented in Table 1. Thirty subjects were male. The average age was 64.8 ± 9.6. All subjects were right‐handed.

### 3.2 Linear regression analysis between WMH volumes and TMT‐A performance

WMH volumes located in the occipital lobe showed a significant association with TMT‐A (*β* = .266; *p* = .040), controlling for age and gender. Relevant details are presented in Table 2.

**Table 2:** Linear regression analysis between regional WMH volumes and TMT‐A completion time

WMHV in cerebral ROIs	TMT‐A	
	*β*	*p*
Frontal WMH	.050	.667
Temporal WMH	.072	.540
Parietal WMH	.097	.402
Occipital WMH	.266	.040*
Subcortical WMH	−.018	.877

*Note*: Beta values (*β*) are presented. Adjusted for age, gender.

Abbreviations: TMT‐A, Trail Making Test Part A; WMH, white matter hyperintensity; WMHV, white matter hyperintensity volume.

* *p* < .05.

### 3.3 Linear regression analysis between gray matter volumes and TMT‐A performance

There was no significant correlation between modulated gray matter maps and TMT‐A performance.

### 3.4 Linear regression analysis between tract average FA and TMT‐A performance

**Figure 3:**
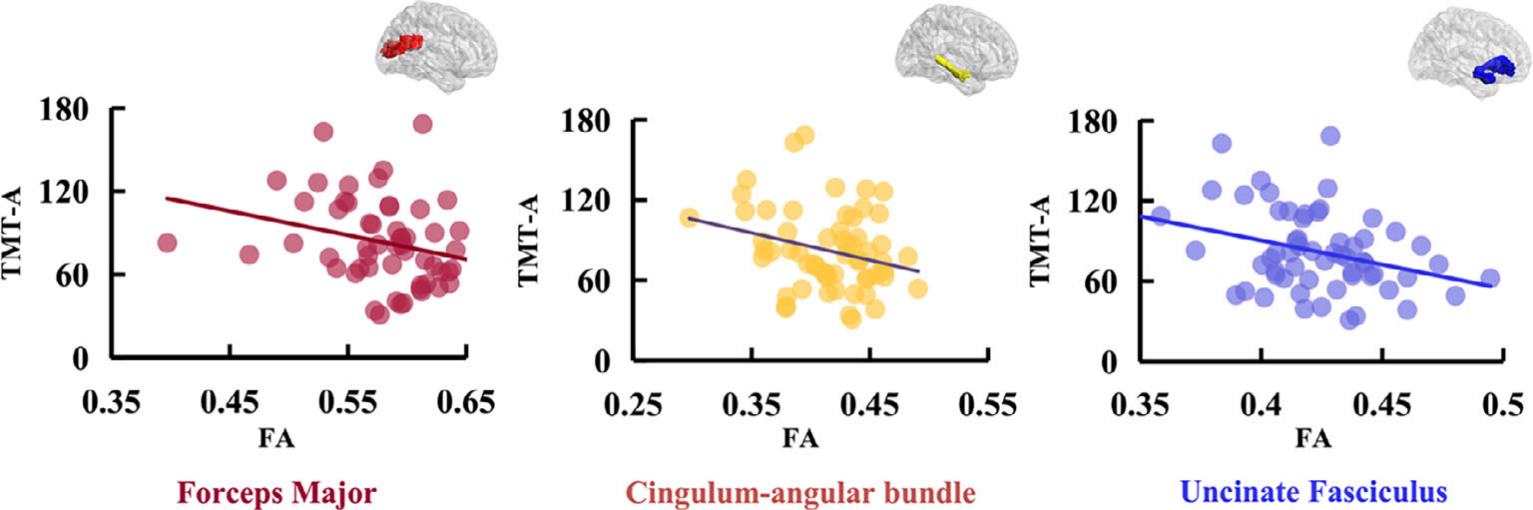
The correlation between the average FA of white matter tracts and TMT‐A completion time. The results of linear regression analysis showed that TMT‐A had a close association with FA in FMajor (*β* = −.259; *p* = .028), right Cab (*β* = −.232; *p* = .045), and right UNC (*β* = −.288; *p* = .010). Cab, cingulum—angular bundle; FA, fractional anisotropy; FMajor, forceps major; TMT‐A, Trail Making Test Part A; UNC, uncinate fasciculus

The average FA values of the constructed 18 tracts are summarized in Table S1. The 18 tracts were reconstructed based on the DTI of each subject. Figure 2 displays the reconstructed tracts from a representative subject. The results of linear regression analysis are presented in Table 3. TMT‐A had a close association with FA in FMajor (*β* = −.259; *p* = .028), right Cab (*β* = −.232; *p* = .045), and right UNC (*β* = −.288; *p* = .010). Figure 3 showed the correlations between the average FA in three significant tracts and TMT‐A completion time.

**Table 3:** Linear regression analysis between tract average FA and TMT‐A completion time

Tract_average FA	TMT‐A			
	*β*		*p*	
FMajor	−.259		.028*	
FMinor	−.152		.184	
	*Left*	*Right*	*Left*	*Right*
ATR	−.046	−.093	.689	.431
Cab	−.203	−.232	.089	.045*
Ccg	−.154	−.085	.182	.469
CST	−.194	−.141	.088	.221
ILF	−.099	−.074	.418	.554
SLFp	−.108	−.033	.388	.784
SLFt	−.112	−.234	.366	.052
UNC	−.081	−.288	.490	.010*

*Note*: Beta values (*β*) are presented. Except for the FMajor and FMinor, all other tracts were reconstructed for the left and right hemisphere.

Abbreviations: ATR, anterior thalamic radiation; Cab, cingulum angular bundle; Ccg, cingulum‐cingulate gyrus bundle; CST, corticospinal tract; FMajor, forceps major; FMinor, forceps minor; ILF, inferior longitudinal fasciculus; SLFp, superior longitudinal fasciculus‐parietal; SLFT, superior longitudinal fasciculus‐temporal; UNC, uncinate fasciculus. Adjusted for age, gender.

* *p* < .05.

### 3.5 Linear regression analysis between fALFF and TMT‐A performance

**Figure 4:**
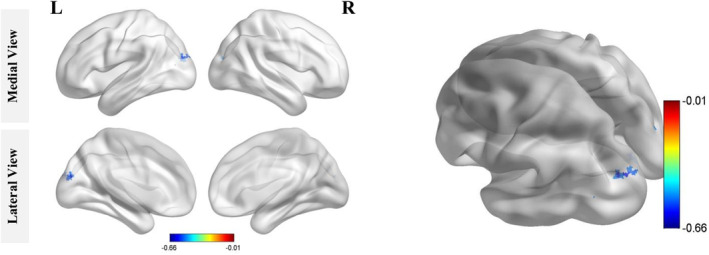
Clusters of fALFF values negatively correlated with TMT‐A performance (FDR corrected). In rs‐fMRI analyses, significantly relevant regions mainly clustered in the occipital lobe, including part of the superior occipital gyrus and middle occipital gyrus (FDR corrected, *p* < .05). fALFF, fractional amplitude of low‐frequency fluctuations; FDR, false discovery rate; TMT‐A, Trail Making Test Part A

We found that fALFF values mainly clustered in the left occipital lobe (Figure 4) and negatively correlated with TMT‐A performance after FDR correction. Specifically, subjects with lower fALFF values had longer TMT‐A completion times. Detailed clusters information is provided in Table 4.

**Table 4:** The summary of brain regions in the significant clusters

Regions	Peak MNI			Voxels
	*x*	*y*	*z*	
BA 18/Cuneus_L/Occipital_Sup_L	−18	−90	21	24
Occipital lobe /Occipital_Mid_L	−33	−78	9	4
Cerebellum_8_R	36	−51	57	3
Left cerebellum/cerebellum posterior lobe	−12	−45	−42	2

*Note*: The results of whole brain fALFF analysis, corrected by false discovery rate (FDR) method, adjusted for age and gender, *p* < .05.

Abbreviations: BA, Brodmann's area; Cerebellum_8_R, right cerebellum 8; Cuneus_L, left cuneus; MNI, Montreal Neurological Institute; Occipital_Sup_L, left superior occipital gyrus; Occipital_Mid_L, left middle occipital gyrus.

### 3.6 Hierarchical multiple regression analysis

As shown in Table 5, age (Model 1) explained 23.8% of the variance in psychomotor processing speeds. FA in FMajor (Model 2) increased the variance to 27.8% (*R* ^2^Δ *=* 5.0%, *p* = .047). Regional fALFF values as a predictor (Model 3) significantly increased the variance to 54.9% (*R* ^2^Δ *=* 27.1%, *p* < .001).

**Table 5:** The summary of hierarchical regression models to predict TMT‐A performance

Psychomotor processing speed				
Model	Independent variables	Adjusted *R* ^2^ (Δ)	*βj*	*p*
*Model 1*		23.8		<.001
	Age		.501	<.001
*Model 2*		27.8 (5.0)		.047
	Age		.553	<.001
	FA in FMajor		−.231	.047
*Model 3*		54.9 (27.1)		<.001
	Age		.402	<.001
	FA in FMajor		−.200	.030
	Regional fALFF values		−.540	<.001

*Note*: Adjusted *R* ^2^ (explanation of variance in the percentages) and standardized beta values (*βj*) were presented for significant findings. Incremental explanations of variance are shown in brackets as delta (Δ) of adjusted *R* ^2^ in percentage. *p* < .05.

Abbreviation: FMajor, forceps major.

## 4 DISCUSSION

**Figure 5:**
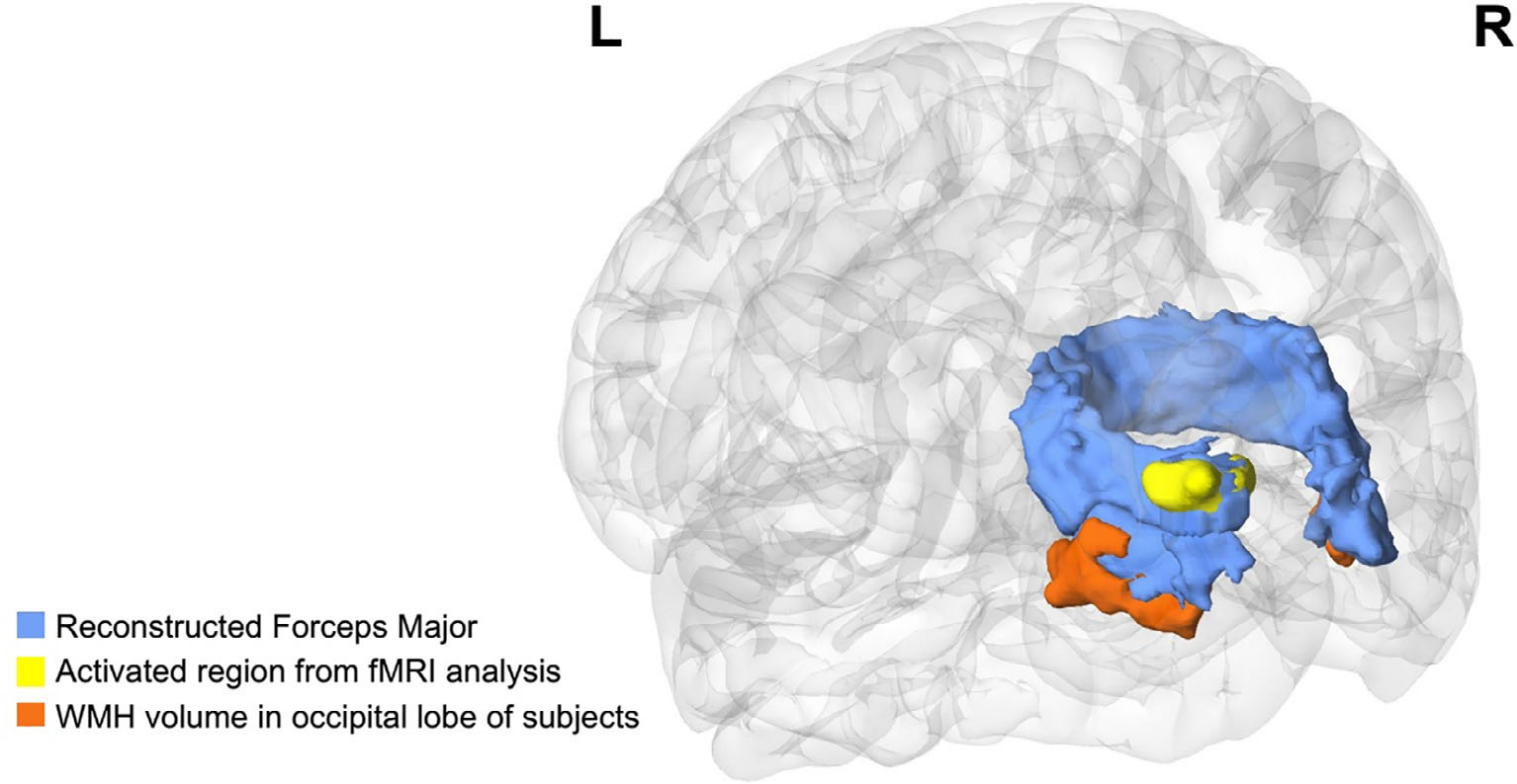
Spatial overlapping diagram of the analyses results between processing speed and macrostructure, microstructure, and brain resting‐state activities. Blue represents the reconstructed FMajor correlated with the TMT‐A completion time; yellow represents the significant cortical region from linear regression analysis between the TMT‐A completion times and resting‐state brain activity; orange represents average WMH distribution in the occipital lobe of the subjects. FMajor, forceps major; TMT‐A, Trail Making Test Part A

We performed a combined analysis of macrostructure, microstructure, and cortical activity to explore neural mechanisms of psychomotor processing speed changes in elderly individuals with WMH. We found that TMT‐A completion times were associated with WMH volume, the mean tract FA and resting‐state brain activity, suggesting that structural and functional degeneration contributes to age‐related decline in cognitive function. Interestingly, the results from three modalities converged in the occipital lobe, with precise spatial overlap (Figure 5). In addition, hierarchical multiple regression analysis revealed that FA in FMajor and local fALFF could predict TMT‐A completion time. These results provide a comprehensive understanding of the association between WMH and psychomotor processing speed alterations during aging, which supported the aforementioned WMH‐Tract‐Function‐Behavior link theory.

We found that the higher the occipital WMH burden, the higher the TMT‐A score, which reflected a lower psychomotor speed. Recent studies reported that lower psychomotor speeds were associated with a higher frequency of WM lesion in the parieto‐occipital lobe (Atwi et al., 2018; Foster‐Dingley et al., 2016; Hirsiger et al., 2017). It was also reported that a greater WMH burden in the posterior WM led to poorer visual search performance, which is a domain of psychomotor processing speed (Davis et al., 2009; Marquine et al., 2010). Considering that visual searches are a key stage in trail‐making tests, these results are consistent with our study.

Second, we found that the integrity of several major WM tracts was related to TMT‐A completion times. As mentioned, microstructural integrity of the WM tracts impacts on brain activity in the regions that connect the tracts. However, the impairments on specific fibers could not be accurately located to specific fibers using common voxel‐based statistical methods (Duering et al., 2014). While this can be improved using tract‐based spatial statistics (TBSS), it remains a method for the detection of local alterations considering such approach creates a mean FA map of the centers of all fiber bundles by filling the FA value from the nearest relevant tract centre for each skeleton voxel (Smith et al., 2006). Moreover, as stated in Bach et al. (2014) and Wang, Luo, Mok, Chu, and Shi (2016), this approach lacks an explicit tract representation and limited anatomical specificity, because it overlays the significant results upon the skeleton voxels. To better assess tract‐level degeneration, we used a novel tract reconstruction technique based on global probabilistic fiber tracking (section 2.4.3). We found that the mean FA of FMajor, UNC, and Cab was correlated with TMT‐A performance.

The FMajor connects bilateral occipital cortex, and links the parietal lobe and the visual cortex (Caminiti, Ghaziri, Galuske, Hof, & Innocenti, 2009; Hofer & Frahm, 2006). This involves connecting the dorsal visual pathway, which is vital for spatial information analysis (Ceschin et al., 2015). It has been shown that the FMajor plays an essential role in regulating the efficiency of visual attention (Niogi, Mukherjee, Ghajar, & McCandliss, 2010; Park et al., 2008). Impaired integrity of the FMajor affects the conduction efficiency of visual information transduction. For example, a previous case report found that patients with focal hemorrhages in the FMajor showed dysfunction in their ability to manipulate visuospatial information and orientation (Tamura et al., 2007).

The UNC is considered as an association fiber that connects the frontal, temporal and subcortical structures. A significant association between UNC and information processing speeds is frequently reported (Diao et al., 2015; Kern et al., 2015). Specifically, the impaired integrity of UNC was related to poor performance in the visuospatial task (Metzler‐Baddeley, Jones, Belaroussi, Aggleton, & O'Sullivan, 2011). Notably, our results showed that the right but not left UNC had a significant correlation with TMT‐A performance. This was in line with the previous findings on the functional lateralization of the UNC (MacPherson et al., 2017), suggesting that the left UNC is prone to speech memory, and the right UNC correlates with spatial memory (Kern et al., 2015; Metzler‐Baddeley et al., 2011).

The function of Cab remains blurred, but its anatomic position is close to the visual association cortex in the occipital lobe (Silvanto, Muggleton, Lavie, & Walsh, 2009). Previous studies have shown that the right angular gyrus (AG) is likely to subserve orienting spatial attention ability (Chambers, Payne, Stokes, & Mattingley, 2004; Chechlacz, Rotshtein, & Humphreys, 2012; Yin et al., 2012). It is possible that the right Cab involves visual information processing, contributing to trail‐making tasks.

Although previous studies have demonstrated a heavier WMH burden associated with lower tract FA (Seiler et al., 2018). We also verified that WMH affects the integrity of the whole tract (WMH part and NAWM part) by extracting the average FA of the WMH positive part of the tracts (lesion‐FA) and the remaining (normal appearing white matter FA, NAMW‐FA). These results were in line with previous studies and are summarized in Tables S2 and S3.

Thirdly, we used resting‐state fMRI to explore the correlation between changes in psychomotor speed and abnormal cortical function activities in aging, and its relationship with structural analyses. Interestingly, the significant cortical region derived from our fMRI analysis was also located in the occipital lobe (in the superior occipital gyrus) and was precisely connected with the FMajor (Figure 4). In the literature, this part of the dorsal visual pathway strongly modulates attention, orientation (Blankenburg et al., 2010; Madden et al., 2002), and spatial frequency, contributing to the integration of complex visual information (Blankenburg et al., 2010). Dysfunction in this brain region may damage visual–spatial integration and deteriorate TMT‐A.

Finally, by summarizing the results from different modalities, we found that the WMH burden disrupted the integrity of the forceps major and abnormal cortical activities in the occipital lobe were significantly associated with lower psychomotor processing speeds. Furthermore, the results derived from different modalities displayed favorable spatial correspondence (Figure 4). The forceps major passes through the WMH lesions, which were located near the posterior horn of the lateral ventricles. The areas where brain activities are associated with TMT‐A completion time were directly connected by the forceps major. These findings not only highlight the importance of occipital brain structures and functions for psychomotor processing speeds, but provide strong support for the WMH‐Tract‐Function‐Behavior link (Fjell et al. 2017; Langen et al., 2018; Madden et al., 2017; Seiler et al., 2018). Notably, although assessed only psychomotor processing speeds, this theory can be generalized to other brain functional decline during aging.

Some limitations of our study should be considered. First, as cerebral small vessel disease involves several risk factors, it is difficult to identify matched aging subjects without vascular damage. Second, while a spatial relationship among different brain features was observed, their causality requires further exploration. Longitudinal studies in larger samples are required to validate our findings.

## 5 CONCLUSION

In conclusion, we explored the association between important brain imaging features and psychomotor processing speed decline using a multi‐modality approach. A variety of structural and functional alterations were found related to TMT‐A performance, and the results from different modalities converged in the occipital lobe, highlighting that WMH lesions, FMajor tract disruption, and altered cortical activity are important for the TMT‐A decline during aging. In addition, the precise spatial overlap from different modalities supports the WMH‐Tract‐Function‐Behavior link, which is a useful framework for understanding the association between WMH occurrence and brain functional decline during aging.

## CONFLICT OF INTERESTS

The authors declare that there are no relevant conflicts of interest.

## DATA AVAILABILITY STATEMENT

Research data are not shared.

## Supporting information


**Table S1** The average FA values in the selected tracts
**Table S2** Summary of whole tract voxels, tract specific WMH voxels and ratios
**Table S3** Correlations between tract specific WMH ratios and FA of different portions in tract
**Table S4** Linear regression analysis between lobar WMH volumes and psychomotor processing speed assessments
**Table S5** Linear regression analysis between tract average FA with psychomotor processing speed assessments
**Table S6** Summary of hierarchical multiple regression model to predict psychomotor processing speed
**Figure S1** The distribution range of specific tract average lesion FA and FA in NAWM as well as the paired *t*‐test result (* *p* < .05). ***A***: ***Revised***; ***B***: ***Previous***.
**Abbreviation**: FMajor, Forceps major; rh_cab, the right cingulum‐angular bundle; rh_unc, the right uncinate fasciculus.
**Figure S2** An example of WMH load from a representative subject. a) The FLAIR image in native space; b) The overlapped map of WMH lesion map and FLAIR image. Red represents segmented WMH lesions; the light red part represents WMH lesion in the occipital lobe. c) The corresponding T1‐weighted image.
