# Supplementary material for: Understanding the association between psychomotor processing speed and white matter hyperintensity: A comprehensive multi‐modality MR imaging study
Source: Hum Brain Mapp. 2019 Nov 1;41(3):605–16. doi: 10.1002/hbm.24826 (PMC7267958; doi:10.1002/hbm.24826)
Supplement: Supplementary file 1 — Table S1 The average FA values in the selected tracts Table S2 Summary of whole tract voxels, tract specific WMH voxels and ratios Table S3 Correlations between tract specific WMH ratios and FA of different portions in tract Table S4 Linear regression analysis between lobar WMH volumes and psychomotor processing speed assessments Table S5 Linear regression analysis between tract average FA with psychomotor processing speed assessments Table S6 Summary of hierarchical multiple regression model to predict psychomotor processing speed Figure S1 The distribution range of specific tract average lesion FA and FA in NAWM as well as the paired t‐test result (* p < .05). A: Revised; B: Previous. Abbreviation: FMajor, Forceps major; rh_cab, the right cingulum‐angular bundle; rh_unc, the right uncinate fasciculus. Figure S2 An example of WMH load from a representative subject. a) The FLAIR image in native space; b) The overlapped map of WMH lesion map and FLAIR image. Red represents segmented WMH lesions; the light red part represents WMH lesion in the occipital lobe. c) The corresponding T1‐weighted image. [file HBM-41-605-s001.doc]

**Supplemental materials**

**Fig. S1** The distribution range of specific tract average lesion FA and FA in NAWM as well as the paired t-test result (* *P* < 0.05). ***A: Revised; B: Previous.***

**Abbreviation:** FMajor, Forceps major; rh_cab, the right cingulum-angular bundle; rh_unc, the right uncinate fasciculus.

**Fig. S2** An example of WMH load from a representative subject. a) The FLAIR image in native space; b) The overlapped map of WMH lesion map and FlAIR image. Red represents segmented WMH lesions; the light red part represents WMH lesion in the occipital lobe. c) The corresponding T1-weighted image.
